# Psychometric assessment and validation of the dysphagia severity rating scale in stroke patients

**DOI:** 10.1038/s41598-020-64208-9

**Published:** 2020-04-29

**Authors:** Lisa F. Everton, Jacqueline K. Benfield, Amanda Hedstrom, Gwenllian Wilkinson, Emilia Michou, Timothy J. England, Rainer Dziewas, Philip M. Bath, Shaheen Hamdy

**Affiliations:** 1 Stroke Trials Unit, Division of Clinical Neuroscience, University of Nottingham, Nottingham, UK; 2 Speech and Language Therapy, Nottinghamshire Healthcare NHS Foundation Trust, Nottingham, UK; 3 Vascular Medicine, Division of Medical Sciences and GEM, University of Nottingham, Royal Derby Hospital Centre, Derby, UK; 4 Stroke, Nottingham University Hospitals NHS Trust, Nottingham, UK; 5 GI Sciences, Division of Diabetes, Endocrinology and Gastroenterology, School of Medicine Sciences, University of Manchester and the Manchester Academic Health Sciences Centre, Manchester, UK; 6 Speech Language Pathology, Communication Disorders and Dysphagia, University of Patras, Patras, Greece; 7 Department of Neurology, University Hospital Münster, Münster, Germany

**Keywords:** Stroke, Stroke

## Abstract

Post stroke dysphagia (PSD) is common and associated with poor outcome. The Dysphagia Severity Rating Scale (DSRS), which grades how severe dysphagia is based on fluid and diet modification and supervision requirements for feeding, is used for clinical research but has limited published validation information. Multiple approaches were taken to validate the DSRS, including concurrent- and predictive criterion validity, internal consistency, inter- and intra-rater reliability and sensitivity to change. This was done using data from four studies involving pharyngeal electrical stimulation in acute stroke patients with dysphagia, an individual patient data meta-analysis and unpublished studies (NCT03499574, NCT03700853). In addition, consensual- and content validity and the Minimal Clinically Important Difference (MCID) were assessed using anonymous surveys sent to UK-based Speech and Language Therapists (SLTs). Scores for consensual validity were mostly moderate (62.5–78%) to high or excellent (89–100%) for most scenarios. All but two assessments of content validity were excellent. In concurrent criterion validity assessments, DSRS was most closely associated with measures of radiological aspiration (penetration aspiration scale, Spearman rank rs = 0.49, p < 0.001) and swallowing (functional oral intake scale, FOIS, rs = −0.96, p < 0.001); weaker but statistically significant associations were seen with impairment, disability and dependency. A similar pattern of relationships was seen for predictive criterion validity. Internal consistency (Cronbach’s alpha) was either “good” or “excellent”. Intra and inter-rater reliability were largely “excellent” (intraclass correlation >0.90). DSRS was sensitive to positive change during recovery (medians: 7, 4 and 1 at baseline and 2 and 13 weeks respectively) and in response to an intervention, pharyngeal electrical stimulation, in a published meta-analysis. The MCID was 1.0 and DSRS and FOIS scores may be estimated from each other. The DSRS appears to be a valid tool for grading the severity of swallowing impairment in patients with post stroke dysphagia and is appropriate for use in clinical research and clinical service delivery.

## Introduction

Post stroke dysphagia (PSD) is common affecting upwards of 40% of patients in the hours to days after ictus, and is associated with poor outcome manifest as increased death or dependency, aspiration and pneumonia, and malnutrition^1^. PSD can be identified by screening and clinical bedside assessments, or diagnosed instrumentally (videofluoroscopy, VFS; fibreoptic endoscopic evaluation of swallowing, FEES); screening devices are also in development^2^. The severity of aspiration may be quantified using VFS or FEES, and is typically measured using the penetration aspiration scale (PAS)^3^. Similarly, a number of scales exist for grading the severity of clinical dysphagia based on oral intake, such as the functional oral intake scale (FOIS)^4^, and the dysphagia severity rating scale (DSRS)^5^.

The DSRS is a clinician rated scale that was developed from the dysphagia outcome and severity scale (DOSS)^6^. It grades how severe clinical dysphagia is, by quantifying how much modification is required to fluids and diet, as well as level of supervision, for safe oral intake. The DSRS comprises three subscales that are totalled to give a score ranging from 0 (best) to 12 (worst). The subscales are five-level ordinal assessments of fluid and dietary intake and supervision; each ranges from normal (score 0) to no intake (4) (Table 1).

**Table 1:** Dysphagia Severity Rating Scale (DSRS).

Score	Fluids	Score	Diet	Score	Supervision
**4**	No oral fluids	**4**	Non oral feeding	**4**	No oral feeding
**3**	Pudding consistency	**3**	Puree	**3**	Therapeutic feeding (SALT/trained staff)
**2**	Custard consistency	**2**	Soft, moist diet	**2**	Feeding by third party (untrained)
**1**	Syrup consistency	**1**	Selected textures	**1**	Eating with supervision
**0**	Normal fluids	**0**	Normal	**0**	Eating independently

NB Mashed diet texture recommendations will be rated as for ‘puree’ diet texture.

As with the DOSS, which ranked independence levels according to the Functional Independence Measures (FIM) model and was linked to severity^7^, supervision on the DSRS was also divided into independence levels; however, the DSRS does not require a VFS to be performed. To date, the DSRS has been used in several published trials of PSD^5,8–10^. The DSRS is copyright free and open access for research use. The aim of the present study was to test and describe the validity of the DSRS in patients with recent stroke. Consensual, content, concurrent criterion, and predictive criterion validity, and internal consistency, inter- and intra-rater reliability, sensitivity to change, and minimal clinically important difference were each assessed. Additionally the relationship with the FOIS, a validated dysphagia scale^4^, was examined.

## Methods

### Approvals, informed consent and ethical approval

This validation study of the DSRS used a mix of prospectively-collected data from completed clinical studies and newly collected prospective data; in each case, non-attributable anonymised data were analysed. The completed trials each had national ethics approvals and patients (or surrogates) had given written informed consent, this covering subsequent data analyses; an individual patient data metanalysis has already been published using the three pilot trials. For survey data, the University of Nottingham Faculty of Medicine Research Ethics Committee assessed that a full review by the committee was not indicated as the requests were distributed via professional networks; participation in the surveys was voluntary and anonymous and all data collection was performed in accordance with relevant guidelines and regulations set out by the University. Clinical audit data were collected by members of the clinical team and did not need research ethics approval. The authors will share a subset of anonymised individual patient trial data with the international VISTA Collaboration^11^.

### Validation

Multiple approaches were taken to validate the DSRS including determining consensual, content, concurrent criterion and predictive criterion validity^12^. Additionally, internal consistency, inter- and intra-rater reliability, sensitivity to change, and minimal clinically important difference (MCID) were determined.

### Data sources

Validation assessments used data from all trials and unpublished studies that are currently known to have used the DSRS as an outcome. These included raw data from published trials of pharyngeal electrical stimulation (PES)^5,8–10^, an individual patient data meta-analysis of the first three of these PES trials^13^, and unpublished studies (NCT03499574, NCT03700853). All studies involved patients with acute and/or sub-acute stroke.

Consensual and content validity were assessed from an anonymous survey sent to 20 UK-based Speech and Language Therapist (SLT) experts with experience of working with adults with acquired dysphagia. Relevant additional information was provided to the respondents regarding the background and purpose of the scale, and what patient group it was designed for. Similarly, to establish the MCID, an anonymous survey was distributed to a number of UK professional networks of SLTs with experience of working with adults with acquired dysphagia.

### Consensual validity

This is the validity of a test determined by its general acceptance in the community of users, or by the number of users who judge it to be valid. Consensual validity was assessed by asking respondents to rate 5 scenarios using the DSRS, as recently used in validation of the International Dysphagia Diet Standardisation Initiative (IDDSI) functional diet scale^14^. Scenarios required respondents to rate recommendations of full amounts of oral intake, minimal and consistent oral trials, liquid only diets and accompanying levels of supervision. Respondents were asked to provide additional comments at the end of the survey. Excellent or good agreement were considered acceptable.

### Content validity

This refers to the extent that a test includes all aspects of its construct, including relevance and comprehensiveness. Relevance was assessed using the content validity index (CVI), an indicator of inter-rater agreement that asks experts to appraise how relevant items are;^15,16^ it is particularly appropriate to use on instruments that have scales with multiple items^15,16^. Experts considered the relevancy (score 1 for not relevant, to 4 for highly relevant) for each item on each sub-scale. Item (I-CVI) and scale (S-CVI) level indices were calculated according to Polit^15,16^. In parallel, the subscales were assessed for comprehensiveness and whether their wording was clear.

### Concurrent criterion validity

This demonstrates how well DSRS correlates with other stroke-related clinical and radiological measures taken at the same timepoint. These included radiological aspiration (penetration aspiration score, PAS by VFS^3^), swallowing (Toronto bedside swallowing screening test [TOR-BSST]^17^, using the sum of the 14 components rather than just the dichotomous pass/fail score; and FOIS^4^), neurological impairment (National Institutes of Health stroke scale, NIHSS), disability (Barthel index, BI^18^), dependency (modified Rankin scale, mRS^18^), and quality of life (EuroQoL 5-dimension 3-level, EQ-5D-3L; EuroQoL visual analogue scale, EQ-VAS^19^). Associations were performed at all available timepoints, typically at baseline, and on and after treatment, using Spearman’s correlation coefficient.

### Predictive criterion validity

This demonstrates how well DSRS at baseline correlates with the stroke-related clinical and radiological measures assessed at a later timepoint; the measures are those as identified immediately above for concurrent criterion validity, and analysed using Spearman’s correlation coefficient.

### Internal consistency

This assesses how well the components of the scale relate to each other and is a measure of scale reliability. The interrelation between scores from the three subscales were assessed using Cronbach’s alpha^20^. Data sources were the STEPS, Vasant and PHAST-TRAC trials, and anonymised clinical audit data from a stroke ward as determined by a Speech and Language Therapist (JB) and Research Practitioner (AH).

### Inter/intra-rater reliability

These are the degree of agreement among raters, and among repeated measurements by one rater, respectively. Inter-rater and intra-rater reliability was performed by JB and AH using the same audit data as used for internal consistency. Both measures of reliability were assessed using the inter-class correlation (ICC).

### Sensitivity to change

This is also known as responsiveness^21^ and refers to how well an instrument identifies longitudinal changes, in a proportionate manner^16^. Changes in the DSRS during the rehabilitation phase after stroke, i.e., from study baseline to final follow-up, were assessed using data from the STEPS trial.

### Minimal clinically important difference (MCID)

The MCID is the minimum difference in a score that is considered valuable and changes patient management^22^. MCID was assessed in three different ways through assessment of statistical distribution (both half standard deviation and standard error of mean), anchor, and consensus through a survey^23–25^. Data for analysis of statistical distribution and anchor methods came from the STEPS trial and an individual patient data meta-analysis of three pilot trials of PES^9,13^. The survey involved UK-based SLTs. The survey was sent to a number of professional networks and it was up to the discretion of the network administrators whether the survey was forwarded.

### Relationship between DSRS and FOIS

The DSRS and FOIS measure overlapping aspects of clinical dysphagia although they have an opposite direction of severity. Their relationship and interconversion were determined through mapping equivalent levels and using data from studies that measured both in parallel. Where a range of values was estimated the median of these is given.

### Statistical analyses

In addition to the specific analyses detailed above, standard approaches were used to present results as number (%), median [interquartile range, IQR] or mean (standard deviation, SD).

## Results

### Trial individual patient data

Four trials of pharyngeal electrical stimulation after stroke have been performed where DSRS was recorded: Jayasekeran, Vasant, STEPS and PHAST-TRAC^5,8–10^. Data on DSRS and other clinical and radiological measures were available at baseline and variously at days 2, 14, 30 and 90. The clinical characteristics of patients by baseline DSRS are shown for these studies (Supplementary Table I), note for all supplementary tables, please see online resource. The mean age was 71 (SD 12) years with 109 (38%) female, mean onset to randomisation of 21 (SD 17) days; the most common clinical syndrome was partial-anterior circulation, 92 (43%) and just 3 (1%) patients had a posterior syndrome; 211 (85%) participants had an ischaemic stroke and 38 (15%) an intracerebral haemorrhage. A ceiling effect was noted at baseline with 139 (48%) patients having a maximum DSRS score of 12. Increasing dysphagia impairment, assessed using the DSRS, was significantly associated with time from onset to randomisation, worse neurological deficit (NIHSS), stroke type, dependency (modified Rankin scale), disability (Barthel index), swallow screening (component score on TOR-BSST), radiological aspiration (PAS) and non-oral feeding state (Supplementary Table I).

### Consensual validity

As Speech and Language Therapists (SLTs) are the primary clinicians who treat dysphagia in the UK, anonymous surveys were sent to 20 invited UK based SLT clinicians. Between eight and ten respondents rated each scenario. Seventy percent of respondents had 10+ years’ experience. The areas of expertise of the respondents was: stroke (5), head and neck (2), dementia (1), other (2). Consensus was excellent (100%) for recommendations of full oral intake; moderate (78%) to low (56%) for minimal oral trials of liquids (e.g. 5 sips) and solids (e.g. 5 tsps.) respectively, and high (89%) and moderate (78%) for consistent oral trials of liquids (e.g. 100 ml) and solids (e.g. half portions of diet) respectively (Supplementary Table II). Consensus was excellent for scoring liquid-only  fluids (100%) but not the accompanying diet component of this scenario (63%) which means this component, overall, had a moderate consensus. Supervision scores were high (80–100%) for full oral intake, high (89%) for minimal oral trials and moderate (67%) for consistent oral trials (Supplementary Table II). Respondents’ comments requested clarification on how to score consistent amounts of oral trials and liquid diets.

### Content validity

Ten of the 20 invited UK-based SLTs responded to the anonymous survey. This is an acceptable number of expert views for undertaking content validation of an instrument^15^. All but two components of the DSRS sub-scales had “excellent” relevance (I-CVI > 0.90); “pudding consistency” was good and “selected textures” was fair (Table 2). At a scale level, both the fluid and food scale achieved an S-CVI/Ave rating of 0.84 (good) and the supervision scale a rating of 0.96 (excellent). Expert feedback regarding wording and comprehensiveness are given in Supplementary Table III; many of these related to the lack of mention of IDDSI^26^ in the DSRS definitions, a point we address in the Discussion.

**Table 2:** Content validity of DSRS sub-scales assessed by 10 UK speech and language therapists.

Item	Rating 3 or 4 (N of 3, 4)	I-CVI	Rating	S-CVI	Rating
***Fluids sub-scale***					
No oral fluids	9 (1, 8)	0.90	Excellent	0.84	Good
Pudding consistency	7 (3, 4)	0.70	Good		
Custard consistency	8 (1, 7)	0.80	Excellent		
Syrup consistency	8 (1, 7)	0.80	Excellent		
Normal fluids	10 (2, 8)	1.00	Excellent		
***Diet sub-scale***					
Non oral feeding	9 (1, 8)	0.90	Excellent	0.84	Good
Puree (mashed)	10 (0, 10)	1.00	Excellent		
Soft, moist diet	9 (1, 8)	0.90	Excellent		
Selected textures	5 (3, 2)	0.50	Fair		
Normal diet	9 (0, 9)	0.90	Excellent		
***Supervision***					
No oral feeding	9 (2, 7)	0.90	Excellent	0.96	Excellent
Therapeutic feeding	10 (0, 10)	1.00	Excellent		
Feeding by third party	9 (0, 9)	0.90	Excellent		
Eating with supervision	10 (0, 10)	1.00	Excellent		
Eating independently	10 (0, 10)	1.00	Excellent		

Interpretation of I-CVI: Excellent >0.78; Good > 0.60–0.78; Fair >0.40- < 0.60 15.

Interpretation of S-CVI: Excellent >0.90; Good >0.80- < 0.90^15^.

S-CVI is average of I-CVI in sub-scale.

### Concurrent criterion validity

Data were available for all four trials^5,8–10^. In the largest (STEPS), DSRS at baseline and weeks 2 and 13 was associated significantly and in appropriate directions with measures, at the same time points of aspiration (PAS using VFS), swallowing (TOR-BSST), disability (Barthel index) and dependency (modified Rankin scale) (Table 3). At 2 weeks post randomisation, DSRS was also associated with impairment (NIHSS). DSRS was not related to quality of life measures at 13 weeks post randomisation. The three sub-scale components of the DSRS (fluids, diet and supervision) were also each associated significantly with aspiration at all three time points. Similar magnitudes of associations were seen in the smaller studies of Jayasekeran^5^ and Vasant^8^ (Table 3) although associations did not always reach significance in these studies^8^. Overall, associations were stronger between DSRS and measures of swallowing and aspiration then with global measures of impairment (NIHSS), disability (BI) and dependency (mRS).

**Table 3:** Concurrent criterion validity - Relationships between DSRS and clinical and radiological assessments at a variety of timepoints in trials of pharyngeal electrical stimulation. (Spearman’s rank correlation coefficient).

DSRS*	Measure	Outcome	Range of Values	Timing (weeks)	N	Median (IQR)	rs	P
**STEPS**^9^								
Total score	DSRS	Dysphagia	0 to 12	0	154	7 (8)	—	—
				2	131	4 (5)	—	—
				13	106	1 (3)	—	—
	VFS-PAS	Aspiration	1 to 8	0	154	4.71 (3.66)	**0.488**	**<0.001**
				2	126	3.27 (3)	**0.387**	**<0.001**
				13	95	2.29 (2.93)	**0.398**	**<0.001**
	TOR-BSST	Swallowing	0 to 14	0	154	1 (3)	**−0.167**	**0.038**
				2	127	2 (10)	**−0.459**	**<0.001**
				13	103	6 (13)	**−0.520**	**<0.001**
	NIHSS	Impairment	0 to 42	0	150	9 (10)	0.020	0.81
				2	131	8 (10)	**0.301**	**<0.001**
				13	106	5 (8)	0.117	0.23
	BI	Disability	0 to 100	0	151	20 (40)	**−0.279**	**0.001**
				2	131	25 (60)	**−0.517**	**<0.001**
				13	106	65 (65)	**−0.407**	**<0.001**
	mRS	Dependency	0 to 5	0	151	4 (1)	**0.179**	**0.028**
				2	131	4 (2)	**0.382**	**<0.001**
				13	106	4 (2)	**0.279**	**0.004**
	EQ-VAS	QoL	0 to 100	13	87	58 (35)	−0.149	0.17
	EQ-5D-3L	QoL	−0.5 to 1.0	13	95	−0.04 (0.489)	−0.109	0.29
Fluids	VFS-PAS	Aspiration	1 to 8	0	154	4.71 (3.66)	**0.498**	**<0.001**
				2	126	3.27 (3)	**0.374**	**<0.001**
				13	95	2.29 (2.93)	**0.362**	**<0.001**
Diet				0	154	4.71 (3.66)	**0.402**	**<0.001**
				2	126	3.27 (3)	**0.416**	**<0.001**
				13	95	2.29 (2.93)	**0.371**	**<0.001**
Supervision				0	154	4.71 (3.66)	**0.417**	**<0.001**
				2	126	3.27 (3)	**0.236**	**0.008**
				13	95	2.29 (2.93)	**0.343**	**0.001**
Jayasekeran^5^								
Total score	DSRS	Dysphagia	0 to 12	0	28	5.5 (11)	—	—
				2	28	2.5 (5)	—	—
	VFS-PAS	Aspiration	1 to 8	0	28	4.5 (3)	0.345	0.073
				2	28	4 (3)	0.146	0.46
	BI	Disability	0 to 20	0	28	6 (4)	−0.340	0.077
				2	28	14 (7)	−0.273	0.16
Vasant^8^								
Total score	DSRS	Dysphagia	0 to 12	0	36	8 (8)	—	—
				2	34	3 (8)	—	—
				13	32	1 (3)	—	—
	VFS-PAS	Aspiration	1 to 8	0	18	3.50 (4)	**0.551**	**0.018**
				2	15	3 (2)	**0.537**	**0.039**
				13	10	1 (2)	0.159	0.66
	NIHSS	Impairment	0 to 42	0	36	11.50 (11)	0.142	0.41
				2	33	6 (7)	**0.378**	**0.030**
				13	26	4 (5)	0.301	0.14
	BI	Disability	0 to 100	0	36	21.50 (39)	−0.017	0.92
				2	34	37.50 (50)	**−0.400**	**0.019**
				13	27	65 (58)	−0.258	0.20
	mRS	Dependency	0 to 5	0	35	4 (1)	−0.030	0.86
				2	33	3 (2)	**0.359**	**0.040**
				13	27	2 (2)	0.311	0.11
**PHAST-TRAC**^10^								
Total score	DSRS	Dysphagia	0 to 12	0	69	12 (0)	—	—
				0.3	60	10.5 (2.5)	—	—
				13	52	5.1 (5.2)	—	—
	FOIS	Dysphagia	1 to 7	0	69	1 (0)	ND	ND
				0.3	60	1.8 (1.3)	**−0.955**	**<0.001**
				13	52	4.3 (2.6)	**−0.978**	**<0.001**

*DSRS range is 0–12 for total score, and 0–4 for subscales.

BI: Barthel index; DSRS: dysphagia severity Rating scale; EQ-5D-3L/HUS: EuroQoL-5 dimension-3 level as health utility scale; EQ-VAS: EuroQoL-visual analogue scale; FOIS: functional oral intake scale; mRS: modified Rankin scale; NIHSS: National Institutes of Health Stroke Scale; PAS: penetration aspiration scale^3^; Richmond agitation and sedation scale (RASS)^29^; VFS: videofluoroscopy.

ND: Not done - all participants had DSRS = 12 and FOIS = 1 at baseline^27^^.^

DSRS was strongly negatively correlated with FOIS at day 2 and week 13 in the PHAST-TRAC trial (Table 3); the association could not be performed at baseline since all participants had a DSRS of 12/FOIS of 1 as part of the trial’s inclusion criteria^27^.

### Predictive criterion validity

Using data from the STEPS trial, baseline DSRS was associated with radiological aspiration (VFS PAS) at 2 and 13 weeks; and swallowing (TOR-BSST), disability (BI) and dependency (mRS) at 2 weeks (Table 4). There was no association with impairment (NIHSS), or quality of life (EQ-5D-3L, EQ-VAS). The three DSRS sub-scale components (fluids, diet and supervision) at baseline were also each associated significantly with radiological aspiration at 2 and 13 weeks.

**Table 4:** Predictive criterion validity - Relationships between DSRS at baseline with clinical and radiological assessments on or after treatment in trials of pharyngeal electrical stimulation. (Spearman’s rank correlation coefficient).

Trial	DSRS*	Measure	Outcome	Range of values	Timing (weeks)	N	Median (IQR)	rs	P
STEPS^9^	Total score	VFS-PAS	Aspiration	1 to 8	2	126	3.27 (3)	**0.461**	**<0.001**
					13	95	2.29 (2.93)	**0.419**	**<0.001**
		TOR-BSST	Swallowing	0 to 14	2	127	2 (10)	**−0.252**	**0.004**
					13	103	6 (13)	−0.131	0.19
		NIHSS	Impairment	0 to 42	2	132	8 (10)	0.094	0.28
					13	106	5 (8)	−0.010	0.92
		BI	Disability	0 to 100	2	132	25 (59)	**−0.281**	**0.001**
					13	107	65 (65)	−0.176	0.070
		mRS	Dependency	0 to 5	2	132	4 (2)	**0.177**	**0.042**
					13	110	4 (2)	0.048	0.62
		EQ-5D-3L	QoL	−0.5 to 1.0	13	95	−0.04 (0.489)	−0.075	0.47
		EQ-VAS	QoL	0 to 100	13	87	58 (35)	0.070	0.52
	Fluids^†^	VFS-PAS	Aspiration	1 to 8	2	126	3.27 (3)	**0.445**	**<0.001**
					13	95	2.29 (2.93)	**0.394**	**<0.001**
	Diet^†^				2	126	3.27 (3)	**0.365**	**<0.001**
					13	95	2.29 (2.93)	**0.351**	**<0.001**
	Supervision^†^				2	126	3.27 (3)	**0.388**	**0.001**
					13	95	2.29 (2.93)	**0.342**	**0.001**
Jayasekeran^5^	Total score	VFS-PAS	Aspiration	1 to 8	2	28	4 (3)	−0.220	0.26
		BI	Disability	0 to 20	2	28	14 (7)	−0.303	0.12
Vasant^8^	Total score	VFS-PAS	Aspiration	1 to 8	2	16	3 (4)	−0.058	0.83
					13	11	1 (2)	−0.169	0.62
		NIHSS	Disability	0 to 42	2	33	6 (7)	0.245	0.17
					13	27	4 (4)	0.104	0.61
		BI	Disability	0 to 100	2	34	38 (50)	−0.242	0.17
					13	28	65 (56)	−0.113	0.57
		mRS	Dependency	0 to 5	2	33	3 (2)	0.098	0.59
					13	28	2 (2)	0.099	0.62

*DSRS range: 0–12 for total score; 0–4 for subscales.

^†^Associations between post-treatment DSRS and outcome measures at subsequent timepoints.

BI: Barthel index; DSRS: dysphagia severity Rating scale; EQ-5D-3L/HUS: EuroQoL-5 dimension-3 level as health utility scale; EQ-VAS: EuroQoL-visual analogue scale; FOIS: functional oral intake scale; mRS: modified Rankin scale; NIHSS: National Institutes of Health Stroke Scale; PAS: penetration aspiration scale; VFS: videofluoroscopy.

Associations between baseline DSRS and post-treatment measures in the trials of Jayasekeran and Vasant were not statistically significant. It was not possible to assess the relationship between baseline DSRS and post treatment FOIS in the PHAST-TRAC trial since all participants had a baseline DSRS score of 12^27^.

### Internal consistency

The interrelation between the scores from the three subscales, at various timepoints, were assessed using Cronbach’s alpha^20^ using data from STEPS, Vasant and PHAST-TRAC trials^8–10^. Internal consistency was “Good” at baseline, varied between “Good” and “Excellent” over the first two weeks, and “Excellent” at 12 weeks (Supplementary Table IV). Similarly, audit of clinical data by JB and AH revealed “Excellent” consistency between the subscales (Supplementary Table V).

### Inter/intra-rater reliability

DSRS was scored in 31–58 hospitalised stroke patients by JB and AH. The inter-rater reliability was “Excellent” for DSRS with intra-class correlation (ICC) = 0.955 (95% confidence intervals 0.925, 0.973); similarly, the intra-rater reliability was “Excellent” (Table 5). Assessments within the subscale were mostly excellent with one good and one moderate result.

**Table 5:** Intra- and inter-rater reliability for DSRS and subscales assessed using the intra-class correlation. Each rater scored data on two occasions separated by a month.

	Comparison	Scale	ICC	Interpretation
Inter-rater	1 (n = 58)	DSRS	0.955 (0.925, 0.973)	Excellent
		Fluids	0.837 (0.740, 0.900)	Good
		Diet	0.985 (0.974, 0.991)	Excellent
		Supervision	0.952 (0.921, 0.971)	Excellent
Inter-rater	2 (n = 31)	DSRS	0.929 (0.859, 0.965)	Excellent
		Fluids	0.721 (0.501, 0.855)	Moderate
		Diet	0.982 (0.965, 0.992)	Excellent
		Supervision	0.958 (0.915, 0.979)	Excellent
Intra-rater	1 (n = 41)	DSRS	1.00 (1.00, 1.00)	Excellent
		Fluids	1.00 (1.00, 1.00)	Excellent
		Diet	1.00 (1.00, 1.00)	Excellent
		Supervision	1.00 (1.00, 1.00)	Excellent
Intra-rater	2 (n = 31)	DSRS	1.00 (1.00, 1.00)	Excellent
		Fluids	1.00 (1.00, 1.00)	Excellent
		Diet	1.00 (1.00, 1.00)	Excellent
		Supervision	1.00 (1.00, 1.00)	Excellent

Interpretation: Excellent >0.90; good >0.75–0.90; moderate 0.50–0.75; poor <0.50.

### Sensitivity to change

**Figure 1:**
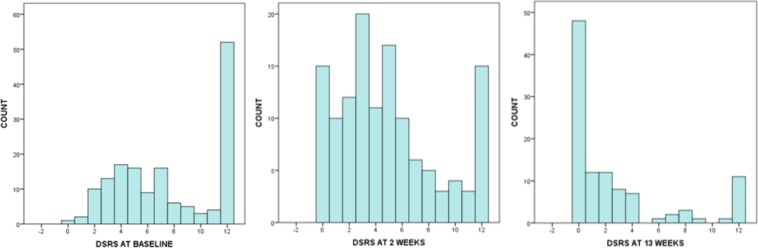
Histograms of distributions of Dysphagia Severity Rating Scale from STEPS trial. At baseline (n = 154), mean 7.6 (3.8), median 7.0^8^, mode 12; at week 2 (n = 131), mean 4.9 (3.7), median 4.0^5^, mode 3; at week 13 (n = 106) mean 2.7 (3.9), median 1.0^3^, mode 0.

DSRS scores were sensitive to spontaneous recovery for patients with acute/subacute PSD, declining during follow-up in STEPS with modal values of 12, 3 and 0 at weeks 0 (baseline), 2 and 13 respectively (Fig. 1). Similarly, the median (7, 4, 1) and mean (7.6, 4.9, 2.7) values declined at the same timepoints. As with VFS-PAS, DSRS was sensitive to treatment with pharyngeal electrical stimulation in a meta-analysis of three pilot trials being 1.7 points lower (p = 0.040) in the PES group as compared with the control group^13^. In contrast, the STEPS trial was neutral for the effect of PES on VFS-PAS and there was no difference in DSRS scores between treatment groups^9^.

### Minimal clinically important difference (MCID)

The survey was based on 84 responses from UK based SLTs, the majority of whom had more than 10 years’ experience. It was not possible to estimate the number that received the survey therefore response rate could not be calculated. MCID varied between 0.3 and 2.5 with all three approaches - statistical, anchor and survey - identifying a MCID of 1.0 as being important (Supplementary Table VI).

### Relationship between DSRS and FOIS

FOIS could be extrapolated from DSRS scores (Supplementary Table VII); however, some combinations of DSRS subscale scores are incongruent from a clinical perspective (e.g. use of thickened fluids when taking a normal diet) and so these have no equivalent FOIS value. Conversely, DSRS could be estimated from FOIS although in most cases it was not possible to determine subscale results since the subscales of supervision and fluids above level 3 are not scored on the FOIS (Supplementary Table VIII).

The PHAST-TRAC trial recorded both DSRS and FOIS at multiple post-randomisation timepoints (days 2, 4, 6, 8, 10, 30 and 90)^10^. The frequency of paired scorings is shown in Supplementary Table IX. The inverse nature of DSRS and FOIS is noted and percentages match the estimated equivalents in Supplementary Tables VII and VIII.

## Discussion

This comprehensive assessment of the DSRS suggests that it is a valid tool for grading dysphagia severity (based on oral intake and supervision requirements) in patients with post-stroke dysphagia. Using data from four randomised controlled trials and 2 surveys, the DSRS was found to exhibit consensual validity, content validity, concurrent criterion validity, predictive criterion validity and internal consistency. Once operationalisation of scoring for certain feeding scenarios was undertaken, inter- and intra-rater reliability were “excellent” when used in a clinical audit, and the minimal clinically important difference approximated to 1 unit irrespective of the method of estimation. The DSRS was sensitive to change during the natural resolution of dysphagia seen through the sub-acute and rehabilitation phases after stroke, and in response to treatment with pharyngeal electrical stimulation in some trials. The intrinsic relationship between DSRS and FOIS allowed these two dysphagia scales to be mapped to each other.

The main strength of this study is the large number and variety of detailed validations performed. We also provide data on minimal clinical important difference and a means for interconverting the DSRS and FOIS. Second, much data came from two phase III trials (STEPS, PHAST-TRAC) rather than just a number of smaller studies. Third, patients with a range of post-stroke severity were included in these validations, with mild-to-moderate patients coming from three trials^5,8,9^ and more severe ones from one^10^. Fourth, a large amount of clinical and radiological outcome data were available. This showed that overall, the DSRS was highly correlated with another clinical measure of dysphagia severity (FOIS). Measures of aspiration (VFS-PAS) and swallowing (TOR-BSST) were more strongly correlated than global measures of impairment, disability, and dependency (although these still showed some significant correlations) and (perhaps surprisingly) the DSRS was not correlated with a generic health status measure of quality of life.

There are a number of caveats to the study. First, although all trial protocols gave some guidance on how to use the DSRS, it was not the primary outcome measure in any study and was largely done according to local practice. Hence, the DSRS scores, whilst prospectively collected, are potentially less accurate than could be achieved with formal training and this was reflected in the consensual validity exercise and respondents’ accompanying comments. In particular, there was less consensus for scoring patients on oral trials and liquid diets, as noted previously^14^. There was also less consensus on assigning supervision scores for patients on consistent amounts of oral trials, i.e. respondents found it easier to score supervision for patients either on full oral intake or limited trials. It is important that raters routinely using the DSRS clearly specify supervision level when making recommendations following the clinical bedside assessment. In the updated version of the DSRS (in Table 6), we provide rules for scoring supervision, including assigning diet, fluid and supervision scores for oral trials.

**Table 6:** Updated Dysphagia Severity Rating Scale incorporating International Dysphagia Diet Standardisation Initiative (IDDSI) levels^25^.

Score	Fluids	Score	Diet	Score	Supervision
4	No oral fluids	4	Non oral feeding	4	No oral feeding
3	IDDSI level 4 - extremely thick	3	IDDSI level 4 - pureed or level 5 - minced & moist	3	Therapeutic feeding (SALT/trained staff)
2	IDDSI level 3 - moderately thick	2	IDDSI level 6 - soft & bite sized	2	Feeding by third party (untrained)
1	IDDSI level 1- slightly thick or level 2 - mildly thick	1	IDDSI level 7 - easy to chew	1	Eating with supervision
0	IDDSI level 0 - thin	0	IDDSI level 7 - regular	0	Eating independently

DSRS supervision score 3 is always chosen when a patient is on limited or consistent oral trials and still requires NG/ PEG tube.

Oral trials are scored from the fluid and diet subscales (i.e. 3 onwards) and can be either trials of food *or* fluid or trials of food *and* fluids.

Second, the DSRS was devised and first used in 2010 and so antedates the 2017 IDDSI scale for determining levels of fluid thickness and modified food textures^26^. Further, DSRS measures different domains from IDDSI. Nevertheless, some comments by respondents in our assessment of content validity commented on the fact that the DSRS does not contain IDDSI terminology regarding wording and comprehensiveness. Going forward, we have proposed a redefinition of the DSRS to reflect IDDSI descriptors (Table 6) and plan to validate this updated scale in due course. Third, although the associations between DSRS and other radiological and clinical measures in the trials of Jayasekeran and Vasant^5,8^ were similar in magnitude to those seen in the STEPS trial, most were statistically non-significant due to their much smaller sample size and so reduced statistical power. This emphasises the importance of having large data sets when performing validation studies of clinical scales. Last, the distribution of DSRS will depend on the population of patients being studied and timing after stroke, and ceiling and floor effects are present at different times after stroke; for example in STEPS, one third of participants had a maximum score of 12 at baseline (reflecting the trial’s inclusion criteria) and a minimum score of zero 13 weeks later after natural resolution of dysphagia; this situation is analogous with other scales used in stroke, e.g. the Barthel Index^28^.

In summary, this study has shown that the 12-level DSRS is robust in terms of consensual, content, concurrent criterion and predictive criterion validity. Further, it shows “good-to-excellent” internal consistency, “excellent” inter- and intra-rater reliability, is sensitive to natural and therapeutic change, and has a minimal clinically important difference of 1 point. However, distribution of scores will depend on patient population and time post-onset. Specific guidance for accurate use of the DSRS is provided in the updated version, which includes corporation of the new IDDSI descriptors. Overall, our results suggest the DSRS is a valid tool for grading the severity of dysphagia in stroke; its ease of use make it relevant for use in clinical service delivery and clinical trials to define baseline dysphagia severity and assess the effect of natural history or therapeutic change.

## Supplementary information

Supplementary Material.
