# Supplementary material for: Psychometric assessment and validation of the dysphagia severity rating scale in stroke patients
Source: Sci Rep. 2020 Apr 29;10:7268. doi: 10.1038/s41598-020-64208-9 (PMC7190822; doi:10.1038/s41598-020-64208-9)
Supplement: Supplementary file 1 — Supplementary Material. [file 41598_2020_64208_MOESM1_ESM.pdf]

Psychometric assessment and validation of the dysphagia severity rating scale in stroke patients

Everton, Lisa F [1,2] MPhil
Benfield, Jacqueline [3] MSc
Hedstrom, Amanda [3] BSc
Wilkinson, Gwenllian [1,4] BMedSci
Michou, Emilia [5,6] PhD
England, Timothy [3] PhD FRCP
Dziewas, Rainer [7] MD
Bath, Philip M [1,4] DSc FMedSci ‡
Hamdy, Shaheen [5] PhD FRCP ‡
‡ Joint senior author

Contact details of corresponding author:

Prof Philip M Bath
Stroke Trials Unit, Division of Clinical Neuroscience
City Hospital Campus, University of Nottingham
Hucknall Road, Nottingham NG5 1PB, UK
Tel: 09 44 115 823 1765
Fax: 00 44 115 823 1767
Email: philip.bath@nottingham.ac.uk

SUPPLEMENTAL MATERIAL

Supplementary Table I. Baseline clinical characteristics in four trials by baseline dysphagia severity rating scale (DSRS) at baseline.

	N	All	DSRS											rs	p-value
	287	8.5 (3.9)	<3	3	4	5	6	7	8	9	10	11	12		
N			22	17	26	20	12	18	10	11	6	6	139	-	-
Age (yr)	287	71 (12)	68	71	73	74	73	76	78	74	80	78	69	-0.093	0.12
Female (%)	287	109 (38)	13.6	35.3	57.7	40	50	44.4	60	27.3	66.7	33.3	34.5	-0.025	0.67
OTR (days)	287	21 (17)	16.5	14.1	12.4	15.2	16.6	18.4	11.10	21	17	19.2	26.11	0.319	**<0.001**
Syndrome (%)	215													-0.007	0.92
TACS		71 (33)	22.7	47.1	24.0	45.0	8.3	16.7	70	54.5	60	0	33.3		
PACS		92 (43)	45.5	23.5	56.0	30.0	58.3	55.6	20	27.3	20.0	83.3	43.5		
LACS		49 (23)	31.8	29.4	20.0	25.0	33.3	27.8	10	9.1	0	16.7	21.7		
POCS		3 (1)	0	0	0	0	0	0	0	9.1	20.0	0	1.4		
NIHSS (/42)	282	12 (7)	8.64	10.7	9.3	10	9.2	8.6	17.7	13.9	11.2	8.7	14	0.304	**<0.001**
Type (%)	249													0.147	**0.020**
IS		211 (85)	95.5	92.9	95	100	50	81.3	100	88.9	66.7	100	80.3		
ICH		38 (15)	4.5	7.1	5	0	50	18.8	0	1.1	33.3	0	19.7		
mRS (/6)	254	4.2 (1.1)	3.54	3.9	3.5	4.0	3.9	4.3	4.6	4.4	4.0	3.0	4.5	0.390	**<0.001**
Barthel index (/100)	215	25.9 (28.3)	32.4	32.7	37.3	28.4	27.2	19.6	8.2	7.6	18.6	33.67	23.9	-0.189	**0.005**
TOR-BSST failed (%)	190	185 (97)	92.3	93.8	100	100	90.9	94.4	100	100	100	100	98.4	0.073	0.31

TOR-BSST (/14) [a]	154	2.4 (3.9)	3.0	2.9	2.9	2.8	1.1	3.5	0.5	2.6	5.0	5.0	1.6	-0.167	**0.038**
PAS (/8)	200	4.7 (2.0)	3.5	3.6	3.4	4.0	4.3	5.4	4.4	4.2	5.2	4.8	5.9	0.475	**<0.001**
Feeding, non-oral (%)	287	205 (71)	18.2	11.8	30.8	40	58.3	61.1	60	90.9	100	83.3	99.3	0.625	**<0.001**

[a] STEPS only

ICH: intracerebral haemorrhage; IS: ischaemic stroke; LACS: lacunar syndrome; OTR: onset to randomisation; PACS; partial anterior circulation syndrome; PAS: penetration aspiration scale; POCS: posterior circulation syndrome; TACS: total anterior circulation syndrome

Supplementary Table II. Consensual validity assessed using consensus for 5 scenarios. Data are number (%) agreeing

Agreement (%)	Fluids	Diet	Supervision
Full oral intake			
1. Mr Smith is having syrup fluids, puree diet and being fed.	10 (100)	10 (100)	8 (80)
2. Miss Brown is having normal fluids and managing most normal foods. However, she is still avoiding certain textures but eats independently.	10 (100)	10 (100)	9 (100)
Minimal oral trials			
3. Mrs Jackson is having 5 sips of custard consistency fluids and 5 tsps. of chilled smooth puddings 3 x day. She has an NG Tube in.	7 (78)	5 (56)	8 (89)
Consistent oral trials			
4. Mr Jones is having half puree meals 3 x day and 100ml syrup fluids 3 x day, he has an nasogastric tube *in situ*, and he is being fed.	8 (89)	7 (78)	6 (67)
Liquid diet			
5. Mrs Ward is on a purely liquid diet of syrup consistency (such as smoothies, fortified drinks), does not require tube feeding and eats independently.	8 (100)	5 (63)	10 (100)

Comment
Respondents' comments reflected uncertainty on how to score oral intake for those on oral trials and liquid diets and how to score supervision for patients on consistent amounts of oral trials.

Supplementary Table III. Content validity: respondents' comments on comprehensiveness and wording

%	Response	Fluids	Solids	Supervision
Comprehensive	Yes	30	20	60
	No	40	40	40
	Comment-only given	30	40	-
Wording	Clear	50	30	80
	Unclear	20	60	20
	Comment-only given	30	10	-

Comments from respondents
Terminology – most respondents noted that the labels were not updated to reflect the terminology used by the International Dysphagia Diet Standardisation Initiative (IDDSI)[25], which has been adopted by the UK (where respondents were based).

Need for more detailed descriptors – some respondents felt that more detail was needed to define terms, for example, "selected textures", or that a description of bolus cohesiveness/food consistency should be included. Respondents also noted that some terms were subjective.

Missing items – respondents felt that additional levels would be helpful to include, e.g. having a separate group for pre-mashed or mashable foods; sub-dividing supervision into distant versus close supervision; including a category of slightly thick fluids (as present in IDDSI).

Supplementary Table IV. Internal consistency assessed using Cronbach's alpha for three trials

**Day**	**0**	**2**	**4**	**6**	**8**	**14**	**90**
STEPS [9]							
N	154	ND	ND	ND	ND	131	106
Alpha	0.89 (0.66, 0.78)					0.88 (0.63, 0.77)	0.92 (0.72, 0.84)
Interpretation †	Good					Good	Excellent
Vasant [8]							
N	28	ND	ND	ND	ND	28	27
Alpha	0.88 (0.54, 0.84)					0.87 (0.52, 0.83)	0.91 (0.61, 0.88)
Interpretation †	Good					Good	Excellent
PHAST-TRAC [10]							
N	‡	60	46	41	31	ND	53
Alpha		0.88	0.80	0.92	0.91		0.96
Interpretation †		Good	Good	Excellent	Excellent		Excellent

ND: Not done/measured

† Interpretation of alpha: Excellent ≥0.9; Good ≥0.8-<0.9; Fair ≥0.7-<0.8; Unacceptable <0.7

‡ All patients scored 4 in each subscale

Supplementary Table V. Internal consistency assessed using Cronbach's alpha (95% confidence intervals) for audit data

	Speech Therapist		Research practitioner	
Measure	1 (n=58)	2 (n=31)	1 (n=58)	2 (n=31)
Alpha	0.924 (0.814, 0.982	0.919 (0.746, 0.990)	0.943 (0.846, 0.988)	0.951 (0.808, 0.996)
Interpretation	Excellent	Excellent	Excellent	Excellent

Supplementary Table VI. Tabulation of minimal clinically important difference (MCID)

Method		Source	MCID
Statistical distribution	Half standard deviation	STEPS [9]	1.9
		IPD MA [12]	2.0
	Standard error of mean	STEPS [9]	0.3
		IPD MA [12]	0.5
Anchor	Aspiration at week 2	STEPS [9]	2.5
		IPD MA [12]	0.8
	Oral vs non-oral feeding at week 2	STEPS [9]	1.0
Delphi	Number of DSRS scales needed to show change	Survey	1.0
	Number of points change on each scale	Survey	1.0

Delphi used revised version DSRS (Supplementary Table X) to determine MCID

Supplementary Table VII. Conversion of DSRS to FOIS in patients with post-stroke dysphagia

DSRS fluids	DSRS food	DSRS super-vision	DSRS total	Feasible combination	FOIS	Comments	Number (%) in STEPS
4	4	4	12	yes	1	NBM	61 (18%)
4	4	3	11	no			6 (1%)
4	4	2	10	no			0
4	4	1	9	no			0
4	4	0	8	no			0
4	3	4	11	no			0
4	3	3	10	yes	2 or 3	Minimal or consistent oral trials (diet only), still requires NG/ PEG	3 (<1%)
4	3	2	9	no			3 (<1%)
4	3	1	8	no			2 (<1%)
4	3	0	7	no			2 (<1%)
4	2	4	10	no			0
4	2	3	9	yes	2 or 3	Minimal or consistent oral trials (diet only), still requires NG/ PEG	0
4	2	2	8	no			1 (<1%)
4	2	1	7	no			1 (<1%)
4	2	0	6	no			1 (<1%)
4	1	4	9	no			0
4	1	3	8	yes	2 or 3	Minimal or consistent oral trials (diet only), still requires NG/PEG	0
4	1	2	7	no			0
4	1	1	6	no			0

4	1	0	5	no				0
4	0	4	8	no				0
4	0	3	7	no				0
4	0	2	6	no				0
4	0	1	5	no				0
4	0	0	4	no				0
3	4	4	11	no				0
3	4	3	10	yes	2 or 3	Minimal or consistent oral trials (both fluids and diet), still requires NG/ PEG		0
3	4	2	9	no				0
3	4	1	8	no				0
3	4	0	7	no				1 (<1%)
3	3	4	10	no				0
3	3	3	9	yes	2 or 3	Minimal or consistent oral trials (both fluids and diet), still requires NG/ PEG		2 (<1%)
3	3	2	8	yes	4			4 (1%)
3	3	1	7	yes	4			7 (2%)
3	3	0	6	yes	4			2 (<1%)
3	2	4	9	no				
3	2	3	8	yes	2 or 3	Minimal or consistent oral trials (both fluids and diet), still requires NG/ PEG		0
3	2	2	7	yes	5			0
3	2	1	6	yes	5			0
3	2	0	5	yes	5			1 (<1%)
3	1	4	8	no				0
3	1	3	7		2 or 3	Minimal or consistent oral trials (both fluids and diet), still requires NG/ PEG		0
3	1	2	6	yes	6			0
3	1	1	5	yes	6			1 (<1%)
3	1	0	4	yes	6			0
3	0	4	7	no				0

3	0	3	6	no		Cannot have thickened fluids and be on a normal diet (which includes mixed consistencies)	0
3	0	2	5	no		Cannot have thickened fluids on a normal diet (which includes mixed consistencies)	0
3	0	1	4	no		Cannot have thickened fluids on a normal diet (which includes mixed consistencies)	0
3	0	0	3	no		Cannot have thickened fluids on a normal diet (which includes mixed consistencies)	1 (<1%)
2	4	4	10	no			2 (<1%)
2	4	3	9	yes	2 or 3	Minimal or consistent oral trials (fluids only), still requires NG/ PEG	2 (<1%)
2	4	2	8	yes, but exception	4	ONLY for patients on a full liquidised diet, managing without NG/ PEG, CF corresponds to IDDSI “liquidised diet”/ moderately thick fluids	1 (<1%)
2	4	1	7	yes, but exception	4	ONLY for patients on a full liquidised diet, managing without NG/ PEG, CF corresponds to IDDSI liquidised diet/ moderately thick fluids	3 (<1%)
2	4	0	6	yes, but exception	4	ONLY for patients on a full liquidised diet managing without NG/ PEG, CF corresponds to IDDSI liquidised diet/ moderately thick fluids	0
2	3	4	9	no			0
2	3	3	8		2 or 3	Minimal or consistent oral trials (both fluids and diet), still requires NG/ PEG	3 (<1%)
2	3	2	7		4		6 (1%)
2	3	1	6		4		4 (1%)
2	3	0	5		4		4 (1%)
2	2	4	8	no			0
2	2	3	7	yes	2 or 3	Minimal or consistent oral trials (both fluids and diet), still requires NG/ PEG	0
2	2	2	6		5		2 (<1%)

2	2	1	5		5		8 (2%)
2	2	0	4		5		1 (<1%)
2	1	4	7	no			0
2	1	3	6	yes	2 or 3	Minimal or consistent oral trials (both fluids and diet), still requires NG/ PEG	0
2	1	2	5	yes	6		0
2	1	1	4	yes	6		2 (<1%)
2	1	0	3	yes	6		0
2	0	4	6	no			0
2	0	3	5	no		Cannot have thickened fluids on a normal diet (which includes mixed consistencies)	0
2	0	2	4	no		Cannot have thickened fluids on a normal diet (which includes mixed consistencies)	0
2	0	1	3	no		Cannot have thickened fluids on a normal diet (which includes mixed consistencies)	0
2	0	0	2	no		Cannot have thickened fluids on a normal diet (which includes mixed consistencies)	0
1	4	4	9	no			2 (<1%)
1	4	3	8	yes	2 or 3	Minimal or consistent oral trials (fluids only), still requires NG/ PEG	0
1	4	2	7	no			1 (<1%)
1	4	1	6	no			0
1	4	0	5	no			1 (<1%)
1	3	4	8	no			0
1	3	3	7		2 or 3	Minimal or consistent oral trials (both fluids and diet), still requires NG/ PEG	2 (<1%)
1	3	2	6		4		6 (1%)
1	3	1	5		4		10 (2%)
1	3	0	4		4		9 (2%)
1	2	4	7	no			0
1	2	3	6		2 or 3	Minimal or consistent oral trials (both fluids and diet), still requires NG/ PEG	0
1	2	2	5		5		3 (<1%)

1	2	1	4		5		15 (4%)
1	2	0	3		5		19 (5%)
1	1	4	6	no			0
1	1	3	5	yes	2 or 3	Minimal or consistent oral trials (both fluids and diet), still requires NG/ PEG	1 (<1%)
1	1	2	4		6		0
1	1	1	3		6		6 (1%)
1	1	0	2		6		4 (1%)
1	0	4	5	no			0
1	0	3	4	no		Cannot have thickened fluids on a normal diet (which includes mixed consistencies)	0
1	0	2	3	no		Cannot have thickened fluids on a normal diet (which includes mixed consistencies)	0
1	0	1	2	no		Cannot have thickened fluids on a normal diet (which includes mixed consistencies)	3 (<1%)
1	0	0	1	no		Cannot have thickened fluids on a normal diet (which includes mixed consistencies)	3 (<1%)
0	4	4	8	no			2 (<1%)
0	4	3	7	yes	2 or 3	Minimal or consistent oral trials (fluids only), still requires NG/ PEG	0
0	4	2	6	no			0
0	4	1	5	no			0
0	4	0	4	no			0
0	3	4	7	no			0
0	3	3	6		2 or 3	Limited or consistent oral trials (both fluids and diet), still requires NG	0
0	3	2	5		4		1 (<1%)
0	3	1	4		4		2 (<1%)
0	3	0	3		4		2 (<1%)
0	2	4	6	no			0
0	2	3	5		2 or 3	Minimal or consistent oral trials (both fluids and diet), still requires NG/ PEG	0
0	2	2	4		5		0

0	2	1	3		5		7 (2%)
0	2	0	2		5		17 (5%)
0	1	4	5	no			0
0	1	3	4	yes	2 or 3	Minimal or consistent oral trials (both fluids and diet), still requires NG/ PEG	0
0	1	2	3	yes	6		0
0	1	1	2		6		7 (2%)
0	1	0	1		6		13 (3%)
0	0	4	4	no			0
0	0	3	3		2 or 3	Minimal or consistent oral trials (both fluids and diet), still requires NG/ PEG	1 (<1%)
0	0	2	2	yes	7†		1 (<1%)
0	0	1	1		7†		4 (1%)
0	0	0	0		7†		56 (16%)

Notes:

1. DSRS supervision score 3 is always chosen when patient is on limited or consistent oral trials and still requires NG/ PEG tube.
2. Oral trials can be either trials of food *or* fluid or trials of food *and* fluids.
3. Note exception of how to score when a patient is having a full liquid diet only and is managing without an NG/ PEG tube.
4. Frequency: This will depend on the population being studied. DSRS=12 is likely to be common at baseline in dysphagia trials, and DSRS=0 reflects an excellent outcome. Example frequencies are shown for STEPS (all timepoints).

† Represents resolution

Supplementary Table VIII. Conversion of FOIS to median value of total DSRS

FOIS	DSRS			
	Total	Fluids	Food	Supervision
1	12	4	4	4
2	7	0-4	0-4	3
3	7	0-4	0-4	3
4	6	0-3	3-4	0-2
5	5	0-3	2	0-2
6	4	0-3	1	0-2
7	0	0	0	0-2

Supplementary Table IX. Relationship between post-treatment DSRS and FOIS measures in the PHAST-TRAC trial. Data are percentages for each combination of DSRS and FOIS.

		FOIS						
	%	1	2	3	4	5	6	7
DSRS	0					0.5		11.1
	1					0.5	1.6	
	2					0.5	0.5	
	3			0.5		1.6	1.1	
	4				2.1	0.5	0.5	
	5			0.5	1.1	1.6		
	6		0.5			1.1		
	7		1.1	0.5	1.6	0.5		
	8		2.1	1.1	1.1	0.5		
	9	0.5	1.6	2.1	1.6			
	10		4.2	0.5				
	11	2.1	2.6	0.5				
	12	48.7	0.6	0.5				
